# Patient-Reported Outcomes of Bleomycin Sclerotherapy for Low-Flow Vascular Malformations and Predictors of Improvement

**DOI:** 10.1007/s00270-018-1999-8

**Published:** 2018-06-08

**Authors:** S. E. R. Horbach, J. S. van de Ven, P. T. Nieuwkerk, Ph. I. Spuls, C. M. A. M. van der Horst, J. A. Reekers

**Affiliations:** 1 Department of Plastic, Reconstructive and Hand Surgery, Academic Medical Center (AMC), P.O. Box 22660, 1100 DD Amsterdam, The Netherlands; 2 Department of Medical Psychology, Academic Medical Center (AMC), Amsterdam, The Netherlands; 3 Department of Dermatology, Academic Medical Center (AMC), Amsterdam, The Netherlands; 4 Department of Radiology, Division of Interventional Radiology, Academic Medical Center (AMC), Amsterdam, The Netherlands

**Keywords:** Bleomycin, Sclerotherapy, Vascular malformations, Venous malformations, Lymphatic malformations

## Abstract

**Purpose:**

There is paucity of data on patient-perceived outcomes of bleomycin sclerotherapy for low-flow vascular malformations. In this study, the long-term outcomes of bleomycin sclerotherapy were investigated in terms of quality of life (QoL) and patient-perceived changes in health.

**Materials and Methods:**

A cohort of Dutch patients with vascular malformations treated with bleomycin sclerotherapy (June 2010-November 2015) completed a questionnaire evaluating disease symptoms, QoL (Short Form 36), patient-perceived change in health status (Global Rating of Change scales) and treatment satisfaction. QoL was assessed for the patient’s status before and after treatment and was analyzed relative to an age and sex-matched Dutch reference population. Predictive factors associated with QoL and patient-perceived improvement in overall health status were assessed using multivariable linear and logistic regression analyses, respectively.

**Results:**

Seventy-seven patients, with a median follow-up of 22 months, were enrolled. About half of the respondents (49.3%) indicated that they perceived (any form of) improvement in their overall health status. Most often improved were the specific health aspects ‘pain’ (54.5%) and ‘overall severity of symptoms’ (57.1%). No factors were significantly predictive for patient-perceived improvement in health with respect to the vascular malformation. Impairment in work- or study-related activities prior to sclerotherapy was found to negatively impact physical QoL at follow-up (*p* = 0.03).

**Conclusion:**

Approximately half of patients with low-flow vascular malformations indicate an improvement in overall health status following bleomycin sclerotherapy, particularly concerning pain and severity of symptoms. However, most patients only perceived little to moderate improvement to their health and desire further treatment.

## Introduction

Low-flow vascular malformations, defined as lymphatic malformations (LMs), venous malformations (VMs) or combined lymphatic-venous malformations (LVMs) [1, 2], can cause a great variety of complaints ranging from pain to dissatisfaction with appearance, which can dramatically affect the patient’s quality of life (QoL) [3, 4].

With sclerotherapy, the affected vessels can be targeted using a sclerosing agent that causes endothelial dysfunction or destruction and an immunologic response followed by thrombotic occlusion and regression of the vascular malformation vessels [5–7]. Sclerotherapy can be used as a stand-alone therapy or can be performed in combination with other procedures such as surgery or laser therapy [8]. Although many sclerosing agents, such as ethanol, etoxysclerol, polidocanol and OK-432, can be used for this goal [8–13], there is no consensus about which sclerosing agent is superior since well-designed randomized comparative trials have not yet been performed [8, 14]. Bleomycin is a sclerosing agent used for vascular malformations [12, 15], that was originally discovered for its cytotoxic and antibiotic properties [16–18]. As clinicians noticed that bleomycin also has a potent sclerosing effect, and may lead to less severe local adverse events (e.g., swelling, nerve injury) than other sclerosing agents [19–21], it is now one of the most frequently used sclerosing agents for vascular malformations [8]. Previously published observational studies show that bleomycin can reduce the size of both LMs and VMs [12, 15]. However, this is usually measured with non-radiologic assessments of change in size or appearance that are subject to the perception of the physician [12]. Although patients regularly undergo treatment to improve subjective complaints such as their symptoms, cosmetic appearance or their health-related QoL, these outcomes are rarely measured from the perspective of the patient. The objective of this study is therefore to explore the long-term treatment outcomes of bleomycin sclerotherapy from the perspective of the patient by using an existing patient-reported outcome measures (PROM) to measure QoL and patient-perceived changes in health after treatment. Furthermore, we aimed to identify variables that are associated with patient-perceived improvement and health-related QoL.

## Materials and Methods

### Study Design

A retrospective follow-up study was performed to assess patients’ perceptions of changes in QoL and various health aspects following bleomycin sclerotherapy in an academic vascular anomalies center.

### Treatment Procedure

In our center, bleomycin is the sclerosing agent of choice for VMs, LMs and LVMs. Prior to treatment, all vascular malformations were assessed for flow characteristics, location and extent of the lesion using non-contrast-enhanced T1- and T2-weighted magnetic resonance imaging (MRI) and/or ultrasonography. Sclerotherapy procedures were performed under deep sedation by an experienced interventional radiologist (J.R. or R.v.d.B.) in the interventional radiologic suite. The vascular malformation is accessed percutaneously using a 22-gauge winged needle under ultrasonographic guidance. Before injecting bleomycin, local phlebography with a non-ionic contrast medium (iodixanol, 320 mg iodine/ml; Visipaque, GE Healthcare, A.S., Oslo, Norway) is performed, following the digital subtraction angiography (DSA) technique, to exclude any direct communication to the systemic circulation and to image the local anatomy of the vascular malformation. Bleomycin (Bleomedac, 15,000 IU/15 mg) is then selectively injected in the venous or lymphatic dilatations only under direct sonographic guidance. After the injections, a local pressure bandage is applied for 6 h. Six weeks after each treatment session, the interventional radiologist assesses clinical response by evaluating symptom relief and visible improvement of the lesion’s size or appearance upon physical examination. If needed, one or more subsequent treatment sessions may be scheduled. In general, the treatment procedure is terminated when there is no (additional) beneficial effect observed after two consecutive treatment sessions or when the patient is satisfied with the result.

### Study Procedure

#### Patient Selection and Eligibility

Adult patients with low-flow vascular malformations (VMs, LMs or LVMs) who underwent bleomycin sclerotherapy as a stand-alone treatment between June 2010 and November 2015 were identified through the prescription database of the hospital pharmacy. Patients treated with bleomycin for diagnoses other than low-flow vascular malformations were excluded. Eligible patients were mailed or e-mailed with an invitation to complete the questionnaire in October 2016, either online or in hard copy. Reminders were sent after 3 and 6 weeks.

#### Questionnaire

Currently, there are no validated patient-reported outcome measures available for patients with vascular malformations. In order to explore the outcome of bleomycin sclerotherapy from the perspective of the patient, the generic QoL measure Short Form 36-item Health Survey (SF-36), global rating of change scales and a treatment satisfaction scale were incorporated in an online questionnaire (SurveyMonkey Inc, San Mateo, California, USA).

*The SF*-*36* is a generic multidimensional instrument that is composed of 36 items sorted into eight multi-item scales representing: (1) physical functioning (PF); (2) role-functioning physical (RP, assesses the extent to which physical health interferes with work or everyday responsibilities); (3) bodily pain (BP); (4) general health perceptions (GH); (5) vitality (VT); (6) social functioning (SF); (7) role-functioning emotional (RE, extent to which, emotional problems cause limitations in work or daily activities); (8) mental health (MH) [22]. With these domains, summary scores can be calculated for the physical component summary (PCS) score and the mental component summary (MCS) score. In this study, the SF-36 was used to assess patients’ follow-up QoL at the time of completing the survey, and in the absence of baseline data, the SF-36 was also utilized for a retrospective baseline measurement. For this purpose, questions from the SF-36 were rephrased to refer to the period before bleomycin sclerotherapy.

*Global Rating of Change (GRC) scales* were applied to gain insight into the magnitude of patient’s perceived changes that occurred following bleomycin sclerotherapy. Patients rated their perceived change on a 7-point Likert scale ranging from -3 (worst possible deterioration) to +3 (best possible improvement). Domains that were rated were overall health status with respect to the vascular malformation (patient’s impression of ‘overall change’ in health), pain, physical well-being, mobility of affected body part, patient-perceived appearance, overall severity of symptoms, work- or study-related activities, emotional well-being and social activities. Although GRC scales are not specifically validated for vascular malformations, they have been used in diverse patient populations and, overall, clinimetric properties such as face validity and responsiveness have shown to be acceptable [23–25].

*Satisfaction* with the treatment procedure and treatment outcome was rated by the patient on a numeric rating scale from 0 (extremely dissatisfied) to 10 (extremely satisfied).

#### Data Collection from Electronic Patient File

The following patient and treatment characteristics were extracted from the electronic patient file: demographics, type of vascular malformations, size categorized based on the largest diameter of the lesion measured on MRI prior to treatment, symptoms prior to treatment, location, lesion depth, prior treatments, sclerotherapy doses and number treatment sessions, complications and follow-up time.

### Data Analyses

Descriptive data were presented for the patient and treatment characteristics, SF-36 scores, GRC scales and satisfaction scores. Mean scores and standard deviations (SD) were used for SF-36 scores and normally distributed data, and median and interquartile ranges were presented for nonparametric data. A paired *t* test was performed to explore the statistical difference between retrospective baseline SF-36 scores and follow-up SF-36 scores.

The 8 dimensions of the SF-36 score were converted to standard scores (Z-scores) on the basis of the scores of age- and sex-matched norms of a representative Dutch population sample [26]. Z-scores were calculated by dividing the difference between the patients’ SF-36 score and the mean score of the age- and sex- matched reference population by the SDs of the reference population. A Z-score indicates how many SDs the observed SF-36 score falls below or above the score of the reference population. Statistical differences between the mean standard scores of the vascular malformation group and the reference population were assessed by means of a one-sample t test. Kruskal–Wallis tests and post hoc Mann–Whitney U tests with Bonferroni correction were computed to explore statistical differences between subgroups of vascular malformation types, sizes and locations.

Bivariate and stepwise multivariable linear regression analyses, adjusted for age and sex, were performed to identify variables predictive for patients’ follow-up SF 36 scores (MCS and PCS), with and without adjustment for the retrospective baseline scores. The analysis without adjustment for the retrospective baseline measurement would yield predictors of a better QoL in general. The analysis with adjustment for the retrospective baseline measurement would explore the predictors of improvement in QoL.

A logistic regression analysis was performed to assess the influence of pre-defined possible predictors (age during treatment, sex, vascular malformation size, vascular malformation type, location of the vascular malformation and symptoms prior to treatment) on the patient’s global rating of improvement. These variables were entered in the multivariable regression analysis when they yielded *p* values < 0.20 in bivariate analyses. Assumption checks regarding multicollinearity, outliers, normality, linearity and homoscedasticity were performed for the multivariable linear regression models. A two-sided *p* value of 0.05 or less was considered statistically significant, and a 95% confidence interval (CI) was used. Data analyses were performed using Statistical Package for the Social Sciences (SPSS version 24.0; IBM Corp, Armonk, NY, USA).

## Results

### Patient and Treatment Characteristics

**Fig. 1:**
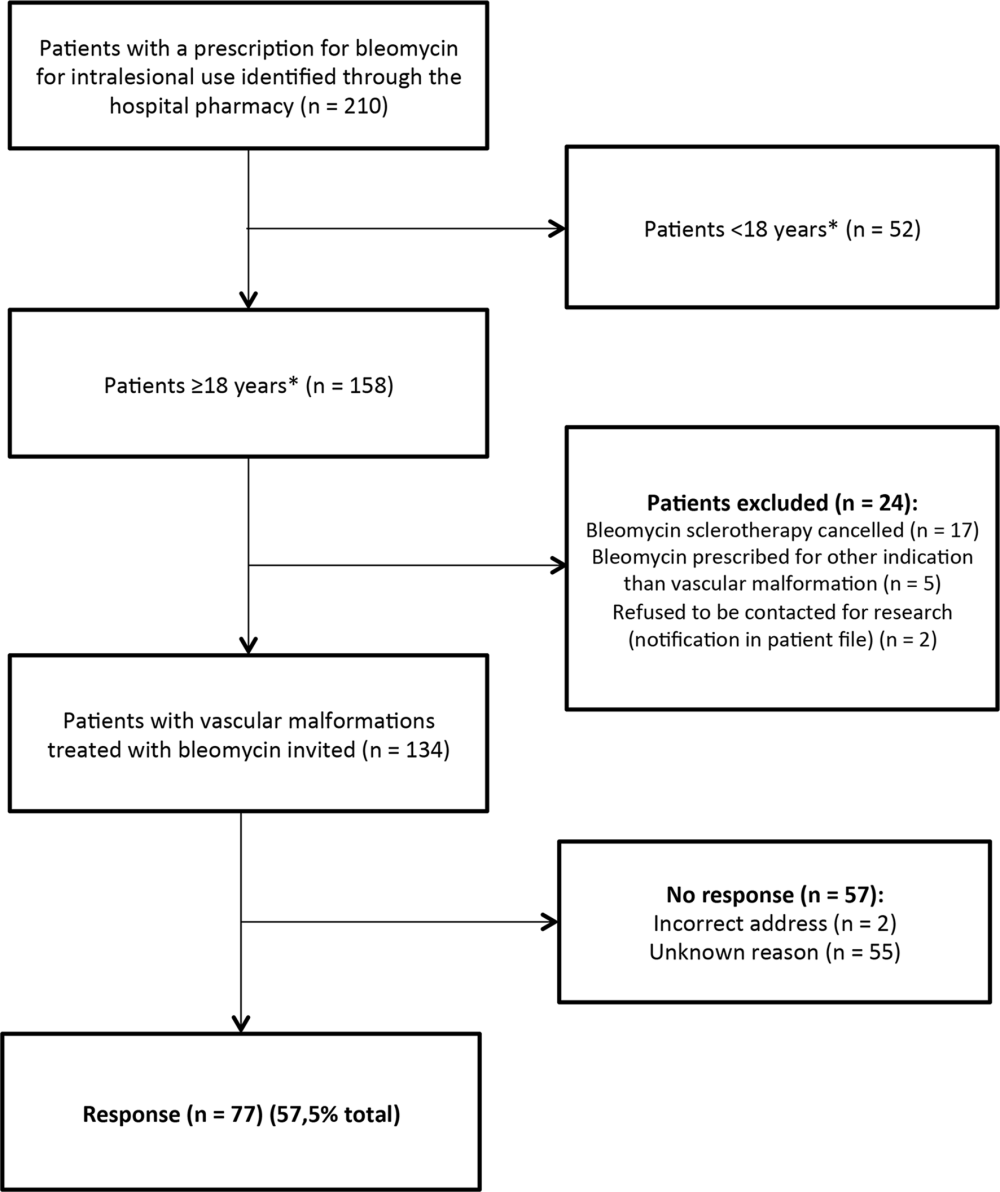
Flowchart of patient enrollment

A total of 134 adult patients who were treated with bleomycin injections for a low-flow vascular malformation were identified from the hospital prescription database and were invited to participate in the study. Seventy-seven of these patients (57.5%) completed the questionnaire (Fig. 1). Patient demographics are shown in Table 1. The median follow-up period from the last treatment session with bleomycin sclerotherapy until completing the questionnaire was 22 months. The majority of patients had VM (81.8%). The most common symptoms reported prior to treatment were pain (74.0%), impaired mobility of the affected body part (49.4%) and dissatisfaction with appearance (37.7%). Five patients underwent treatment procedures other than bleomycin sclerotherapy in the follow-up period: surgery (*n* = 2), laser therapy (*n* = 1) or sclerotherapy with a different sclerosing agent (*n* = 2).

**Table 1:** Patient characteristics

Patients *n* = 77	Median	IQR (25th–75th percentile)
Age (years)	35	26 (24–49.5)
Follow-up time since last bleomycin injection (months)	22	19 (14–32)
Number of bleomycin sclerotherapy sessions	2	1 (1–2)

	Number	Percent (%)
Gender		
Female	50	64.9
Educational level		
No education	1	1.3
High school	9	11.7
Intermediate professional education	28	36.4
Higher professional education (Bachelor’s degree)	27	35.1
Academic education (Master’s degree)	12	15.6
Vascular malformation type		
VM	63	81.8
LM	8	10.4
LVM	5	6.5
VM combined with port-wine stain	1	1.3
Location of vascular malformation		
Head and neck	32	41.6
Trunk	5	6.5
Upper extremities	14	18.2
Lower extremities	26	33.8
Lesion size		
0–5 cm	19	24.7
5–10 cm	10	13.0
> 10 cm	24	31.2
Not reported	25	32.5
Lesion depth		
Skin	1	1.3
Subcutaneous	21	27.3
Muscle	25	32.5
Bone	12	15.6
Not reported	18	23.4
Diagnostic imaging		
Ultrasound	45	58.4
MRI	70	90.9
CT	6	7.8
X-ray	4	5.2
Diagnostic histologic biopsy	8	10.4
Previous therapies		
Sclerotherapy (non-bleomycin)	17	22.1
Surgery	34	44.2
Laser therapy	7	9.1
Compression therapy	22	28.6
Cryotherapy	1	1.3
Radiotherapy	1	1.3
None	19	24.7
Symptoms prior to bleomycin sclerotherapy*		
Pain	57	74.0
Itch	10	13.0
Recurrent bleedings	8	10.4
Recurrent infections	5	6.5
Recurrent phleboliths/localized intravascular coagulation	11	14.3
Impaired mobility of affected body part	38	49.4
Location-specific symptoms**	2	2.6
Fatigue	5	6.5
Impaired physical condition	20	26.0
Impaired in work- or study-related activities	20	26.0
Impaired in social activities	15	19.5
Dissatisfaction with appearance	29	37.7
Emotional/psychological issues	8	10.4
No symptoms	1	1.3

*Question not answered by 2 patients. **Symptoms associated with the location of the vascular malformation (e.g., visual disturbance, swallowing difficulties)

A total of 151 sclerotherapy sessions were performed in the included 77 patients. The number of sclerotherapy sessions ranged between 1 and 6, with a median of 2 treatment sessions. A concentration of 1 mg (~ 1000 IU) per ml bleomycin was generally utilized, for smaller vascular malformations (< 5 cm) a concentration of 3 mg (~ 3000 IU) per ml was used (*n* = 31, 23.5%).The total dose per session ranged from 1 to 18 mg, with a mean dose of 10.18 mg (SD 5.02). The cumulative doses ranged from 2 to a maximum of 90 mg with a mean of 18.34 mg (SD 16.06 mg).

### Patient-Reported Health-Related QoL

The sum scores of the SF-36 scales and subscales are shown in Table 2. Patients’ follow-up SF-36 scores were significantly higher than their retrospective baseline scores on the following scales: the physical composite scale (PCS) (*p* < 0.01), and the subscales physical functioning (PF) (*p* = 0.01), role physical (RP) (*p* = 0.03), BP (*p* < 0.01), social functioning (SF) (*p* < 0.01) and mental health (MF) (*p* = 0.03). The mental composite scale (MCS) was not statistically improved. There were no statistical differences in QoL scores between patients with LMs, VMs and LVMs (PCS *p* = 0.34, MCS *p* = 0.84) and between patients with different malformation sizes (PCS *p* = 0.25; MCS *p* = 0.23). At follow-up, the PCS scores of patients with vascular malformations in the upper and lower extremities were significantly lower, compared to vascular malformation in the head and neck region (*p* < 0.05 and *p* < 0.005, respectively). The MCS scores did not statistically differ between the location subgroups (*p* = 0.18).

**Table 2:** Short-Form-36 (SF-36) scores

	Retrospective baseline SF-36	Follow-up SF-36*	Paired *t* test		
	Mean ± SD	Mean ± SD	Mean of the difference ± SD	95% CI of difference	*p* value
PCS	46.34 ± 10.11	49.19 ± 9.43	2.85 ± 6.58	1.32; 4.39	**0.00**
MCS	50.77 ± 8.56	51.26 ± 7.60	0.49 ± 7.56	− 1.27; 2.25	0.58
Physical functioning	81.28 ± 19.18	84.91 ± 17.42	3.63 ± 11.71	0.95; 6.30	**0.01**
Role physical	71.49 ± 35.85	78.95 ± 34.65	7.46 ± 29.58	0.70; 14.22	**0.03**
Bodily pain	56.46 ± 29.44	68.33 ± 28.68	11.87 ± 20.01	7.30; 16.44	**< 0.001**
Social functioning	75.49 ± 26.78	84.25 ± 20.01	8.77 ± 22.59	3.64; 13.89	**0.001**
Mental health	76.96 ± 13.17	79.56 ± 13.17	2.60 ± 10.20	0.25; 4.94	**0.03**
Role emotional	84.64 ± 32.86	83.77 ± 32.88	− 0.88 ± 30.78	− 7.91; 6.16	0.80
Vitality	66.73 ± 16.11	66.87 ± 18.56	0.13 ± 11.59	− 2.53; 2.80	0.92
General health perceptions	72.36 ± 20.21	73.48 ± 20.29	1.12 ± 11.89	− 1.61; 3.85	0.41

Bold values indicate statistical significance (*p* < 0.05)

*PCS* physical component summary score, *MCS* mental component summary score

*At the time of completing the questionnaire: median follow-up of 22 months after bleomycin sclerotherapy

**Fig. 2:**
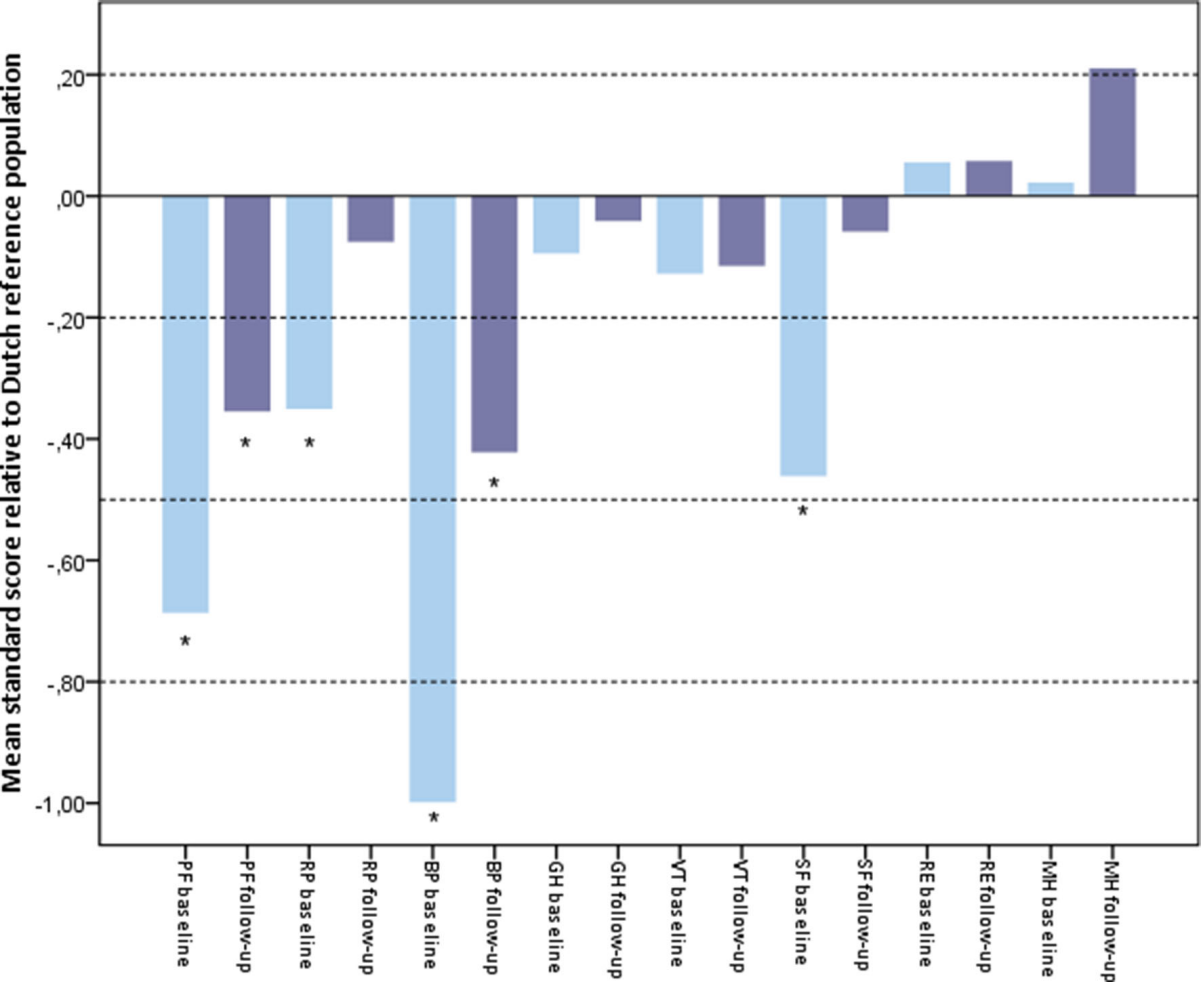
Bar chart of Z-scores of SF-36 domains corrected for age and sex, compared to Dutch reference population mean. The Z-scores or ‘standard mean scores’ indicate how many standard deviations the patient’s score is from the mean of the Dutch reference population. A Z-score of zero (horizontal line) therefore indicates a score conform the reference population. A negative score indicates a lower and a positive score a higher score than the reference population mean.* indicates a significant difference compared with the Dutch reference population. The dotted horizontal lines at standard mean scores of 0.2, 0.5 and 0.8 represent small, moderate and large deviations from the reference population, respectively. Baseline = retrospectively assessed baseline SF-36 score for situation prior to bleomycin sclerotherapy. Follow-up = SF-36 scores after bleomycin sclerotherapy, at the time of completing the questionnaire (median follow-up 22 months). PF = physical functioning, RP = role physical, BP = bodily pain, GH = general health, VT = vitality, SF = social functioning, RE = role emotional, MH = mental health

When these results are analyzed relative to a general Dutch reference population (shown in Fig. 2), the patients’ retrospective baseline scores on physical functioning (PF), role physical (RP), bodily pain (BP) and social functioning (SF) were significantly lower than the age- and sex-matched reference population. At follow-up, only the subscales physical functioning (PF) and bodily pain (BP) remained significantly lower.

### Patient-Reported Global Ratings of Change (GRC)

As shown in Table 3, 49.3% percent of patients indicated that there had been some level of improvement in their overall health status with respect to the vascular malformation following bleomycin sclerotherapy, whereas the remaining patients indicated that their overall health status was unchanged (39.0%) or even deteriorated (11.7%). These ratings did not significantly differ between LMs, VMs, and LVMs (*p* = 0.71), different size categories (*p* = 0.79) or locations (*p* = 0.97). A decrease in pain was noted by 54.5% of the total number of participating patients, and an improvement in overall severity of symptoms was reported by 57.1% of patients. Both mobility of the affected body part and cosmetic appearance were considered improved by 42.9% of patients. Physical well-being (23.4%), work- or study-related activities (31.2%), emotional well-being (22.1%) and social activities (27.1%) improved in a smaller subset of patients. In general, patients rated their improvement on all GRC scales predominantly as ‘a little better’ and only sporadically as ‘very much better,’ indicating that the majority of patients only perceived slight changes to these different aspects of their health.

**Table 3:** Patient-reported scores on the global rating of change (GRC) scales

	Overall health status with respect to vascular malformation		Pain		Physical well-being		Mobility of the affected body part		Appearance/cosmetics		Severity of symptoms		Work- or study-related activities		Emotional well-being		Social activities	
	*n*	%	*n*	%	*n*	%	*n*	%	*n*	%	*n*	%	*n*	%	*n*	%	*n*	%
Improved	38	49.3	42	54.5	18	23.4	33	42.9	33	42.9	44	57.1	24	31.2	17	22.1	21	27.3
Somewhat better	8	10.4	16	20.8	8	10.4	16	20.8	18	23.4	18	23.4	9	11.7	9	11.7	7	9.1
Better	15	19.5	17	22.1	7	9.1	12	15.6	10	13.0	17	22.1	11	14.3	5	6.5	10	13.0
Very much better	15	19.5	9	11.7	3	3.9	5	6.5	5	6.5	9	11.7	4	5.2	3	3.9	4	5.2
No change	30	39.0	23	29.9	49	63.6	31	40.3	34	44.2	21	27.3	46	59.7	56	72.7	53	68.8
Deteriorated	9	11.7	12	15.6	10	13.0	13	16.9	10	13.0	12	15.6	7	9.1	4	5.2	3	3.9
Somewhat worse	4	5.2	4	5.2	6	7.8	9	11.7	9	11.7	8	10.4	5	6.5	3	3.9	2	2.6
Worse	3	3.9	7	9.1	4	5.2	4	5.2	0	0.0	3	3.9	2	2.6	1	1.3	1	1.3
Very much worse	2	2.6	1	1.3	0	0.0	0	0.0	1	1.3	1	1.3	0	0.0	0	0.0	0	0.0

### Treatment Satisfaction

Patients rated their satisfaction with treatment outcome with a median of 6 (IQR 3.25–8.00) and satisfaction with the treatment procedure received a median rating of 7 (IQR 5.00–8.00). A majority of 63.6% of patients expressed a desire for further treatment, mostly because the beneficial effects of bleomycin sclerotherapy did not last or because of clinically significant residual disease.

### Predictive Variables for QoL and Patient-Perceived Improvement

Location in the lower extremities and complaints of fatigue, dissatisfaction with appearance and impairment in work- or study-related activities prior to treatment were significant predictors of having lower follow-up PCS scores, indicating a worse physical QoL. When adjusted for retrospective baseline PCS scores, only the latter factor remained significantly predictive of a lower follow-up PCS score. There were no factors that contributed significantly to the regression models for the MCS follow-up scores (mental QoL) and overall improvement in health status (Table 4).

**Table 4:** Multivariable logistic regression model of overall improvement and linear regression models for SF-36 PCS and MCS follow-up scores

	Multivariable logistic regression			Multivariable linear regression							
				SF-36 PCS				SF-36 MCS			
	Overall patient-reported improvement			Not adjusted for baseline PCS		Adjusted for baseline PCS		Not adjusted for baseline MCS		Adjusted for baseline PCS	
	OR	95% CI	*p* value	*β*	*p* value	*β*	*p* value	*β*	*p* value	*β*	*p* value
Gender (female)	–	–	–	− 0.03	0.73	− 0.05	0.55	0.09	0.49	0.00	0.99
Age	–	–	–	− 0.13	0.15	− 0.12	0.10	− 0.10	0.40	0.16	0.19
*Disease characteristics*											
Size											
0–5 cm	–	–	**–**	–	–	–	–	0.26	**0.04**	**–**	**–**
> 5–10 cm	–	–	**–**	− 0.12	0.28	–	–	–	**–**	**–**	**–**
> 10 cm	–	–	**–**	0.15	0.19	− 0.01	0.89	–	**–**	**–**	**–**
Type											
CVM	–	–	–	0.06	0.49	–	–	–	–	–	–
VM	–	–	–	–	–	–	–	–	–	–	–
LM	–	–	–	–	–	− 0.11	0.13	–	–	–	–
LVM	–	–	–	–	–	–	–	–	–	–	–
Depth/extent											
Superficial (skin)	–	–	–	–	–	–	–	–	–	–	–
Subcutaneous	2.02	(0.64–6.31)	0.23	–	–	–	–	–	–	0.13	0.26
Muscle	–	–	–	–	–	–	–	–	–	–	–
Bone	0.64	(0.16–2.58)	0.53	–	–	–	–	–	–	–	–
Location											
Head/neck	–	–	–	− 0.30	0.17	–	–	− 0.10	0.57	0.19	0.45
Trunk	–	–	–	–	–	–	–	− 0.14	0.28	–	–
Upper extremities	–	–	–	− 0.20	0.25	–	–	− 0.26	0.08	− 0.05	0.80
Lower extremities	–	–	–	− 0.44	**0.03**	–	–	–	–	0.29	0.19
*Symptoms/complaints prior to treatment*											
Pain	–	–	–	− 0.12	0.30	–	–	–	–	− 0.02	0.89
Itch	–	–	–	− 0.15	0.12	− 0.09	0.23	− 0.05	0.67	–	–
Bleeding	–	–	–	–	**–**	–	–	–	–	–	–
Recurrent infections	–	–	–	− 0.14	0.16	− 0.08	0.36	− 0.04	0.75	− 0.11	0.39
Localized thrombosis	–	–	–	–	–	–	–	–	–	–	–
Impaired mobility of the affected body part	–	–	–	− 0.15	0.15	− 0.13	0.12	–	–	0.02	0.88
Location-specific symptoms*	–	–	–	–	–	–	–	–	–	–	–
Fatigue	–	–	–	− 0.35	**0.001**	− 0.16	0.05	− 0.12	0.39	− 0.09	0.46
Impaired physical condition	1.91	(0.60–6.14)	0.27	− 0.09	0.43	–	–	− 0.09	0.51	–	–
Impaired in work or study-related activities	–	–	–	− 0.34	**0.003**	− 0.19	**0.03**	− 0.25	0.06	− 0.11	0.37
Impaired in social activities	–	–	–	− 0.08	0.41	–	–	–	–	–	–
Dissatisfaction with appearance	1.26	(0.44–3.63)	0.67	0.22	**0.04**	0.07	0.36	0.15	0.30	0.05	0.72
Psychological or emotional complaints	–	–	–	–	–	–	–	− 0.11	0.36		

Bold values indicate statistical significance (*p* < 0.05)

*Location-specific symptoms are functional problems associated with the localization of the malformation near sensory organs, gastrointestinal or genito-urethral system, for example visual disturbance or difficulty swallowing. ‘–’*p* > 0.20 in univariable regression analyses

## Discussion

About half of the patients indicated that they noticed any form of improvement in their health status with respect to the vascular malformation after a median follow-up period of 22 months. Particularly pain and overall severity of symptoms were considered improved. However, our data suggest that subtle health changes are generally more likely to occur than drastic improvements. The majority of patients indicate that they desire further treatment.

A possible explanation for the moderate to low patient satisfaction scores could be that the patients’ expectations exceeded the outcomes that could realistically be achieved. In general, long-term complete remission is rarely observed in the management of vascular malformations. Therefore, vascular malformations may be considered as a chronic disease, and patients may require multiple therapeutic interventions during their life course to minimize symptoms and to improve cosmetic appearance. The expectations of patients should be managed accordingly with clear patient–physician communication about the risks, benefits and expectations of treatment.

In retrospect, patients seemed to perceive a better health-related QoL after bleomycin sclerotherapy when comparing to their recalled baseline situation, particularly on the physical subscales such as bodily pain and physical functioning, which would imply that especially psychical QoL may be improved with bleomycin sclerotherapy. Impairment in work- or study-related activities prior to treatment was the only factor that negatively influences physical QoL at follow-up. It is likely that patients with extensive disease, who are already severely impaired in daily life, cannot expect much improvement in this area following bleomycin sclerotherapy.

This study focuses on patient-reported outcomes, an undervalued type of outcome measurement in this patient category, despite the fact that the reasons for initiating therapy are very often patient subjective. However, assessing patients’ perceptions of health and QoL is difficult. This study was limited because of its retrospective study design, which made it impossible to perform a true baseline measurement. Consequently, the retrospective baseline measurement that was performed could have been affected by memory effects or ‘recall bias’ [27]. Another limitation was that we were restricted to a relatively small sample size for our regression analyses, as vascular malformations are uncommon. Nevertheless, we believe that the results of these exploratory analyses are important for guidance in clinical practice, as information about patient-perceived outcomes and predictive factors for improvement was not yet available and could assist physicians in therapeutic decision-making.

Systematic reviews that were largely based on physician-reported outcomes, report a favorable overall response following bleomycin sclerotherapy in 68–100% of treated patients with head and neck vascular malformations [8] and a good to excellent outcome in 87% of VMs and 84% of LMs in any locations of the body [12]. It seems that the patient-perceived improvement in this study is far more modest than the physician-perceived outcomes of improvement that have been published so far. These findings highlight the importance of including standardized patient-reported outcome measures in future studies.

This patient cohort had lower scores on various domains the SF-36 QoL questionnaire compared to the Dutch reference population. These results were largely in line with the findings of other authors [3, 28]. A study of Ono et al. in patients with VM treated with sclerotherapy (unspecified agent) demonstrates a significant improvement in the SF-36 domains role physical, bodily pain and social functioning, which is consistent with our findings [29]. Although this would suggest that change in QoL after treatment in this patient population can be captured by the SF-36, it is important to further investigate the measurement properties of the SF-36 in this patient population with prospectively collected data. For example, it needs to be determined if the SF-36 can adequately measure a change in health status (responsiveness) and which changes in scores are clinically meaningful for patients (interpretability). Furthermore, development and validation of disease-specific PROMs may further benefit outcome measurement in this field.

## Conclusion

Bleomycin sclerotherapy generally leads to moderate patient-reported improvement in health and QoL in about half of treated patients, irrespective of the type, size and location of the lesion. Patient expectations should be managed accordingly.
